# Nutrient availability links mitochondria, apoptosis, and obesity

**DOI:** 10.18632/aging.100505

**Published:** 2012-11-30

**Authors:** Francesca Pintus, Giovanni Floris, Alessandro Rufini

**Affiliations:** ^1^ Medical Research Council, Toxicology Unit/University of Leicester, LE1 1QH, Leicester UK; ^2^ Dipartimento di Scienze della Vita e dell'Ambiente, Università di Cagliari, 09042 Monserrato, Italy

**Keywords:** nutrients, obesity, caloric restriction, apoptosis, mitochondria, aging

## Abstract

Mitochondria are the dominant source of the cellular energy requirements through oxidative phosphorylation, but they are also central players in apoptosis. Nutrient availability may have been the main evolutionary driving force behind these opposite mitochondrial functions: production of energy to sustain life and release of apoptotic proteins to trigger cell death. Here, we explore the link between nutrients, mitochondria and apoptosis with known and potential implications for age-related decline and metabolic syndromes.

## INTRODUCTION

The endosymbiotic theory proposes that mitochondria evolved from an atypical encounter of two prokaryotes that ended up in a symbiotic relationship. One bacterium was phagocytosed but not killed, and became progressively specialized in producing energy through oxidative phosphorylation - it became a mitochondrion. This pivotal event shaped the course of evolution, and was fundamental to the development of eukaryotic cells and metazoa [1, 2]. Progressively mitochondria turned into plastic organelles, specialized in energy production, but they also developed into an efficient cell-killing machine at the core of the apoptotic process. Growing evidence suggests that this mitochondrial apoptotic function is tightly linked to nutrient availability and respiratory efficiency, with important implications for health and aging.

### Mitochondria structure

Millions of years of evolution have substantially modified the initial symbiotic relationship and, while mitochondria specialized in energy production, many regulatory functions have been shifted to the hosting cell. The actual human mitochondrial DNA is a circular molecule of 16Kb and encodes a handful of proteins, tRNAs and rRNAs. Only 13 proteins are encoded by mitochondrial DNA, forming only a small proportion of the components of the electron transport chain (ETC). All the remaining mitochondrial proteins are nuclear encoded [3].

**Figure 1:**
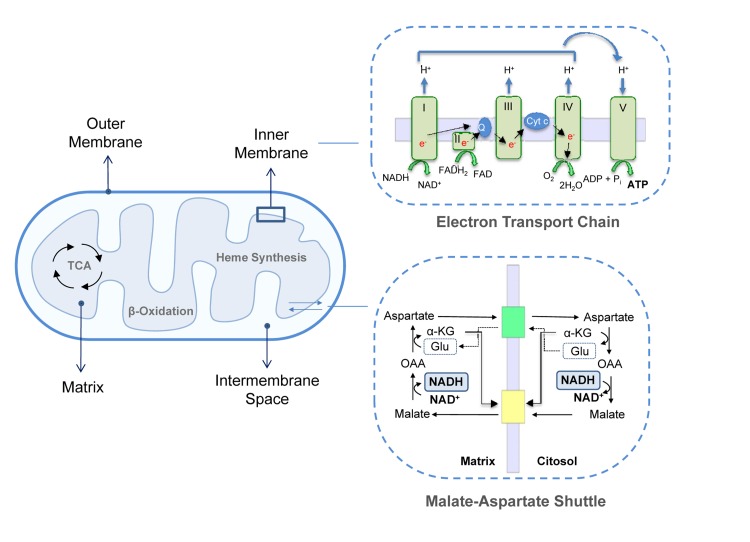
Mitochondrial structure Mitochondrial membranes delimit the IMS and the matrix. This last compartment hosts the mitochondrial metabolic pathways, such as TAC cycle, β-oxidation and heme synthesis. MIM contains ETC complexes and ATP synthase. Complex I, III and IV extrude protons from the matrix in the IMS creating a proton gradient or mitochondrial membrane potential. The retrograde flux of ions promoted by complex V (ATP Synthase) liberates the energy necessary to phosphorylate ADP to ATP; upper inset. Fundamental for mitochondrial homeostasis and function are several exchange carries, such as the malate-aspartate shuttle in which cytosolic oxaloacetate is reduced to malate in a NADH-dependent reaction and malate is then imported in the mitochondrial matrix and oxidized back to oxaloacetate by malate dehydrogenase with the conversion of NAD+ to NADH; lower inset. α-KG, α-ketoglutarate; OAA, Oxaloacetate; Glu, Glutamate, cyt-c, cytochrome c.

Mitochondria are composed of two highly specialized membranes, the **mitochondrial outer membrane** (MOM) and the **inner membrane** (MIM), which possess different structural and functional characteristics. These membranes define two separate mitochondrial compartments: the **mitochondrial matrix** (MM) and the **intermembrane space** (IMS) [4] (Figure 1).

The MOM is smooth and widely permeable to ions and molecules of up to 10KDa. Proteins localized in the MOM are mainly involved in solute exchange, protein import and fatty acid uptake for β-oxidation. Conversely, the MIM is impermeable to most solutes and ions, a property that allows complexes of the ETC to build up the proton gradient required for oxidative phosphorylation. Consequently, specific carriers exist to transport individual molecules across this barrier. Common to all mitochondria are the ADP/ATP carrier, which exports ATP into the cytoplasm while ADP is imported into the matrix, the phosphate transporter and the pyruvate carrier. Other systems transport citric acid cycle intermediates that are used by cells in cytosolic pathways such as fatty acid synthesis and gluconeogenesis. For example, di- and tricarboxylate carriers exchange malate or succinate for HPO_4_^2−^ and citrate and isocitrate for malate, respectively. Since fatty acid β-oxidation occurs in the mitochondrial matrix, long-chain fatty acids are actively transported from the cytoplasm into the mitochondria by the carnitine palmitoyl transferase system of the MIM [5, 6]. Moreover, the MIM contains several transport shuttles used to transport the reducing agents NAD(P)(H) and FAD(H_2_) across the membrane. An example is the malate-aspartate shuttle in which the electrons of NADH are given to oxaloacetate forming malate after which malate is transported across the inner membrane and reoxidized back to oxaloacetate by malate dehydrogenase converting NAD^+^ to NADH in the mitochondrial matrix (Figure 1).

Two distinct domains can be identified in the MIM: the inner boundary membrane (IBM), tightly attached to the MOM, and the cristae, which extend within the matrix space increasing the membranous surface and which enclose ETC complexes and ATP synthase for oxidative phosphorylation. ETC complexes are organized into four large multi-subunits. Complex I (or NADH dehydrogenase) transfers two electrons from NADH, produced by the tri-carboxylic acid (TCA)/Krebs's cycle and β-oxidation, to the lipid-soluble carrier ubiquinone coenzyme-Q (CoQ). Complex II contains succinate dehydrogenase as the only membrane-bound enzyme of the TCA cycle; the electrons are driven from succinate to CoQ using FADH_2_ as coenzyme. In complex III (CoQ - cytochrome c oxidoreductase) two electrons are removed from reduced CoQ and sequentially transferred to two molecules of cytochrome c. Reduced cytochrome c is then oxidized by complex IV (cytochrome c oxidase) that donates electrons to oxygen as the last step of the electron transfer. Complexes I, III and IV, are protons pumps which link electron transfer to the pumping of protons from the matrix into the intermembrane space in order to generate the proton gradient across the MIM. This gradient is then used backwards by ATP synthase, or F_1_F_o_-ATPase to synthesize ATP. This is the major and most efficient source of energy in the cell and, as is well known, the main generator of reactive oxygen species (ROS) [7-9].

The IMS is delimited by the outer and inner membranes and shows a protein composition different from the cytosol and matrix. Its most prominent member is cytochrome c, which has an essential role in the respiratory chain and in the apoptotic signaling pathway since it is released into the cytosol during the apoptotic process [10, 11]. In addition to cytochrome c, other potential apoptotic inducers are present, as well as enzymes such as adenylate kinase and creatine kinase. The MM is the space enclosed by the inner membrane and includes a large range of enzymes that are part of the major metabolic pathways such as the TCA cycle, lipid and amino acid oxidation and urea and heme biosynthesis.

Cellular mitochondrial content can increase through growth and division of pre-existing organelles, a process known as biogenesis and promoted by the peroxisomal proliferative activated receptor-γ co-activator 1α (PGC-1α). PGC-1α enhances transcription of nuclear encoded mitochondrial genes, causing an increase in mitochondrial number and function [12, 13]. In addition, mitochondria are subject to a plastic process of fusion and fission. Fusion generates complex tubular structures with high respiratory efficiency, whereas fission results in fragmented and less efficient mitochondria [12]. In mammals, but with orthologues in Drosophila and yeast, the dynamin-related GTPases mitofusin (Mfn) 1 and 2, and Opa1 are responsible for mitochondrial fusion. Mfn1 and 2 localize to the MOM, whereas Opa1 mediates fusion of the MIM allowing exchange of mitochondrial content. Conversely, dynamin-related protein 1 (Drp1) and fission protein 1 (Fis1) mediate fission: Fis1 localizes to the MOM and serves as an anchor for the recruitment of Drp1, the triggering event of mitochondrial fission [12]. Highly metabolic tissues (such as muscle) show increased fusion, stimulated by PGC-1α-mediated increase of Mfn2 expression. Repression of Mfn2 decreases oxygen consumption and mitochondrial membrane potential [14-16].

Collectively, this chapter highlights mitochondrial specialization as energy hubs and effectors of cellular catabolism.

### Mitochondrial apoptosis

Mitochondria are critical regulators of apoptotic cell death. Apoptosis is a highly conserved and regulated self-suicide mechanism in which there is DNA fragmentation, nuclear condensation and fragmentation into apoptotic bodies [17-23].

There are two main apoptotic pathways: the extrinsic pathway, triggered by cell surface receptor engagement and the intrinsic pathway triggered in response to cellular stress signals and dependent on mitochondria [24]. In the intrinsic pathway, proteins of the Bcl-2-family are crucial determinants of cellular fate. This family comprises antiapoptotic members such as Bcl-2, Bcl-w, Bcl-X_L_ and Mcl-1 and proapoptotic homologs, such as Bax and Bak or the BH3-only proteins Bid, Bim Puma, Noxa Bik and Bmf [25-28]. In response to death stimuli, Bax and Bak oligomerization triggers permeabilization of the MOM, followed by a drop in the membrane potential and release into the cytoplasm of apoptotic effector proteins. The Bax/Bak oligomerization is directly antagonized by antiapoptotic Bcl-2 members, which preserve integrity of the mitochondrial membranes [29, 30]. Finally, BH3-only proteins can displace Bcl-2 proteins from Bax and Bak allowing apoptosis to occur [31, 32]. Perhaps the most relevant protein released from mitochondria during apoptosis is cytochrome c, one of the proteins involved in ETC activity and energy production, which, in the presence of ATP, assembles Apaf-1 and procaspase 9 into the apoptosome. Cleavage and activation of procaspase 9 only occurs, however, when the inhibitory activity of another apoptosome component, IAP, is neutralised by another protein released from mitochondria, Smac/DIABLO [33]. Active caspase 9 is an initiator or apical caspase which in turn activates the executioner caspases 3 and 7 [34]. Briefly, caspases are a family of cysteine proteases that cleave their substrates specifically after aspartic acid residues. All caspases, like caspase 9, exist in cells as catalytically inactive pro-enzymes (zymogens). During apoptosis, initiator caspases activate effector caspases in a cascade of activation in which zymogens are converted into mature enzymes by proteolytic cleavage [35, 36]. Other proteins released during apoptosis include HTRA2, apoptosis inducing factor (AIF) and endonuclase G (endoG), which all play different roles in apoptotic cell death [10, 11, 37, 38].

Mitochondrial dynamics is also altered in apoptotic cells and inhibition of fusion by Bax-mediated repression of Mfn2 seems to be a prerequisite for loss of membrane potential and cytochrome c release. Mitochondria appear fragmented in apoptotic cells and ectopic expression of Mfn2 prevents Bax translocation to the MOM and protects cells from apoptosis [39-42]. Similarly, Drp1 activity is necessary for mitochondrial permeabilization and inhibition of Drp1 increases mitochondrial fusion and prevents release of cytochrome c [28, 40, 43].

In summary, a very complex network has evolved to decide whether a cell should live or die, and mitochondria stand at the heart of this decision. Although perhaps better known for its pathogenic role in some human diseases, such as cancer and neurodegeneration [19, 34, 38, 44-52], apoptosis has important functional roles during development and in the immune response [19]. Strong evidence for this is the lymphopenia, cachexia and nervous system defects affecting mice with a mutation that abolishes the apoptotic function of cytochrome c [53]. Another developmental example is the formation of fingers, a process that involves apoptotic death of cells in the interdigital space.

Forms of apoptosis have been described even in unicellular organisms such as yeast [20, 54], which suggests that regulated cell “suicide” has been an ancient decision in evolution, and probably coevolved with the first endosymbiotic relationship [1, 2]. But while it is easy to understand the function of apoptosis in metazoans, it is be more difficult to imagine its role in unicellular organisms: why would a single cell decide to die and evolve a machinery to achieve this? And, above all, why this pivotal role for mitochondria?

One reason may well be the availability of nutrients. When a colony of unicellular organisms is in good equilibrium with the available extracellular energy resources, proliferation and growth are possible. On the other hand, when resources are limiting, cell death has the effect of reducing colony size to a level where it is again in equilibrium with environmental nutrient supply. In this regard, the ability to die may confer an evolutionary advantage and provide a paradoxical mechanism of survival in conditions where nutrients are limiting. Thus, as mitochondria specialized in energy production, they also became the most obvious sensor of nutrient availability and the most obvious candidate to regulate cell death. It is therefore conceivable that nutrient availability may have been the main evolutionary driving force and ancient stressor that has selected mitochondria for the regulation of apoptosis, which would help explain why some apoptosis-related proteins influence mitochondrial respiration [55-57].

### Mitochondria, apoptosis and nutrients

As mentioned above, in metazoa apoptosis has acquired additional functions important for body shaping during development and during immune responses [19, 58]. However, there remains a strong link between mitochondria, nutrient supply and apoptosis in multicellular organisms.

**Figure 2:**
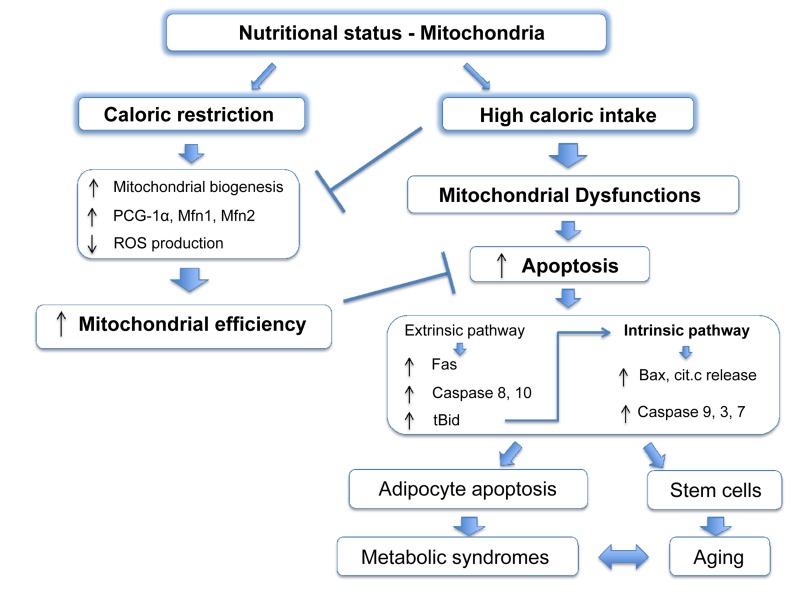
Mitochondrial apoptosis links nutritional status with metabolic syndromes *Caloric restriction* leads to an improvement of the mitochondrial function stimulating mitochondrial biogenesis and fusion, and increasing ETC efficiency with a decreased production of ROS. *High caloric intake* decreases levels of PCG-1α and Mfn2 leading to reduced mitochondrial fusion and compromising organelle functions. Moreover, in obesity apoptotic pathways proteins are upregulated and increased apoptosis has been reported in adipocytes. This cell death is strongly dependent on mitochondria as the extrinsic pathway can also activate the intrinsic pathway by caspase-8 mediated cleavage of the BH3-only protein Bid, resulting in the formation of the active truncated isoform tBid. Genetic depletion of Bid protects against obesity-induced metabolic syndrome. Other cell lines, such stem cells, might suffer a similar mitochondrial dysfunction during obesity and become more sensitive to apoptosis, which in turn, will worsen age and age-related metabolic syndromes.

It is widely accepted that mitochondria play an important role during aging and, like apoptosis, this relationship is conserved from yeast to primates [59-61]. Many mouse models in which mitochondrial function is compromised show accelerated aging [62-68]. It has been disputed whether ROS, the harmful by-products of ETC activity, are important for this outcome [69]. Indeed, in some cases mitochondrial dysfunction correlates with increased ROS production and oxidative damage [63, 65, 66], although other studies failed to see a similar phenotype [70]. Nonetheless, these studies demonstrate that defects in mitochondrial activity aggravate aging. Caloric restriction (CR: reduced food intake without malnutrition), is regarded as one of the most successful approaches to prolong lifespan. From *C. elegans* to mice and primates, reduced food intake improves survival and delays age-associated decline, compared to ad libitum feeding [71]. CR also reduces accumulation of senescent cells in mice [72]. Several molecular pathways have been implicated in this process, such as dampened IGF/insulin signalling and mTOR kinase activity, increased activation of AMPK and reduced ROS production all of which act on or are consequent to mitochondrial function [73, 74]. CR promotes PGC-1α activity and enhances expression of Mfn1 and 2, stimulating mitochondrial biogenesis and fusion [75]. Overall, CR potentiates mitochondrial activity, which is pivotal to its anti-aging outcome [76, 77] (Figure 2).

Although ad libitum feeding is standard laboratory practice, it is unlikely to reproduce animals' natural food intake, which is probably nearer to a regimen of CR. In this regard, since mitochondria evolved to coordinate energy production with food availability, their optimum performance coincides with CR, whereas excess of food intake will compromise mitochondrial energetic capacity [78, 79], probably through mTOR and PGC-1α [74, 75, 77, 80].

Thus, we might envisage a scenario where mitochondria are susceptible to apoptosis as their efficiency of energy production, which is linked to nutritional status, declines. In other words, excess food intake will impair respiratory capacity and prime mitochondria for apoptosis, increasing cellular susceptibility to additional stress. This possibility could also be extended to unicellular organisms, such as yeast [81, 82] and might represent an alternative way to remove cells with inefficient mitochondria when other mechanisms, such as autophagy, are not in place. It is noteworthy that apoptotic proteins levels and cell death are increased in adipocytes of obese humans and rodents, and genetic depletion of the pro-apoptotic BH3-only protein Bid protects from liver steatosis and insulin resistance in high fat diet regimes [83]. An opposite effect, an increase in anti-apoptotic proteins has been suggested to be neuroprotective in aged caloric-restricted mice [84]. In addition, obese individuals have reduced levels of PGC-1α and Mfn2 and obesity results in mitochondrial fragmentation, which is known to reduce mitochondrial energetic efficiency and which has been observed in apoptotic cells [85-87].

In summary, the nutritional imbalance in western diets leads to mitochondrial dysfunction and higher susceptibility to apoptosis with dramatic consequences for metabolic syndromes such as insulin resistance and liver steatosis (Figure 2). It is already known that caloric restriction protects from several stresses [74], and it would be interesting to investigate whether cells isolated from mice on different diets show different susceptibilities to apoptotic cell death via the intrinsic pathway and whether this correlates with the mitochondrial respiratory rate. In particular, adult stem cells could be intriguing candidates for further studies, as they show a particular sensitivity to nutrient availability, and their loss contributes to aging [88-90]. Increased nutrient-mediated susceptibility to cell death may cause stem cell repertoire exhaustion and accelerate aging in obese individuals in a mitochondria-dependent fashion, at least partially explaining the increased apoptotic rates in aged individuals [91-93].

## References

[R1] Huettenbrenner S, Maier S, Leisser C, Polgar D, Strasser S, Grusch M, Krupitza G (2003). The evolution of cell death programs as prerequisites of multicellularity. Mutat Res.

[R2] Kroemer G (1997). Mitochondrial implication in apoptosis. Towards an endosymbiont hypothesis of apoptosis evolution. Cell Death Differ.

[R3] Chen XJ, Butow RA (2005). The organization and inheritance of the mitochondrial genome. Nat Rev Genet.

[R4] Distler AM, Kerner J, Hoppel CL (2008). Proteomics of mitochondrial inner and outer membranes. Proteomics.

[R5] Chen Z, Lash LH (1998). Evidence for mitochondrial uptake of glutathione by dicarboxylate and 2-oxoglutarate carriers. J Pharmacol Exp Ther.

[R6] Indiveri C, Iacobazzi V, Tonazzi A, Giangregorio N, Infantino V, Convertini P, Console L, Palmieri F (2011). The mitochondrial carnitine/acylcarnitine carrier: function, structure and physiopathology. Mol Aspects Med.

[R7] Chance B, Williams GR (1955). Respiratory enzymes in oxidative phosphorylation. I. Kinetics of oxygen utilization. J Biol Chem.

[R8] Hatefi Y (1985). The mitochondrial electron transport and oxidative phosphorylation system. Annu Rev Biochem.

[R9] Murphy MP (2009). How mitochondria produce reactive oxygen species. Biochem J.

[R10] Tait SW, Green DR (2010). Mitochondria and cell death: outer membrane permeabilization and beyond. Nat Rev Mol Cell Biol.

[R11] Parsons MJ, Green DR (2010). Mitochondria in cell death. Essays Biochem.

[R12] Seo AY, Joseph AM, Dutta D, Hwang JC, Aris JP, Leeuwenburgh C (2010). New insights into the role of mitochondria in aging: mitochondrial dynamics and more. J Cell Sci.

[R13] Spiegelman BM (2007). Transcriptional control of energy homeostasis through the PGC1 coactivators. Novartis Found Symp.

[R14] Bach D, Pich S, Soriano FX, Vega N, Baumgartner B, Oriola J, Daugaard JR, Lloberas J, Camps M, Zierath JR, Rabasa-Lhoret R, Wallberg-Henriksson H, Laville M (2003). Mitofusin-2 determines mitochondrial network architecture and mitochondrial metabolism. A novel regulatory mechanism altered in obesity. J Biol Chem.

[R15] Pich S, Bach D, Briones P, Liesa M, Camps M, Testar X, Palacin M, Zorzano A (2005). The Charcot-Marie-Tooth type 2A gene product, Mfn2, up-regulates fuel oxidation through expression of OXPHOS system. Hum Mol Genet.

[R16] De Palma C, Falcone S, Pisoni S, Cipolat S, Panzeri C, Pambianco S, Pisconti A, Allevi R, Bassi MT, Cossu G, Pozzan T, Moncada S, Scorrano L (2010). Nitric oxide inhibition of Drp1-mediated mitochondrial fission is critical for myogenic differentiation. Cell Death Differ.

[R17] Johnson CE, Freel CD, Kornbluth S (2010). Features of programmed cell death in intact Xenopus oocytes and early embryos revealed by near-infrared fluorescence and real-time monitoring. Cell Death Differ.

[R18] Poon IK, Hulett MD, Parish CR (2010). Molecular mechanisms of late apoptotic/necrotic cell clearance. Cell Death Differ.

[R19] Reed JC, Green DR (2011). Apoptosis : physiology and pathology.

[R20] Carmona-Gutierrez D, Ruckenstuhl C, Bauer MA, Eisenberg T, Buttner S, Madeo F (2010). Cell death in yeast: growing applications of a dying buddy. Cell Death Differ.

[R21] Nunez R, Sancho-Martinez SM, Novoa JM, Lopez-Hernandez FJ (2010). Apoptotic volume decrease as a geometric determinant for cell dismantling into apoptotic bodies. Cell Death Differ.

[R22] Tsiatsiani L, Van Breusegem F, Gallois P, Zavialov A, Lam E, Bozhkov PV (2011). Metacaspases. Cell Death Differ.

[R23] van Doorn WG, Beers EP, Dangl JL, Franklin-Tong VE, Gallois P, Hara-Nishimura I, Jones AM, Kawai-Yamada M, Lam E, Mundy J, Mur LA, Petersen M, Smertenko A (2011). Morphological classification of plant cell deaths. Cell Death Differ.

[R24] Galluzzi L, Vitale I, Abrams JM, Alnemri ES, Baehrecke EH, Blagosklonny MV, Dawson TM, Dawson VL, El-Deiry WS, Fulda S, Gottlieb E, Green DR, Hengartner MO (2012). Molecular definitions of cell death subroutines: recommendations of the Nomenclature Committee on Cell Death 2012. Cell Death Differ.

[R25] Garrison SP, Phillips DC, Jeffers JR, Chipuk JE, Parsons MJ, Rehg JE, Opferman JT, Green DR, Zambetti GP (2012). Genetically defining the mechanism of Puma- and Bim-induced apoptosis. Cell Death Differ.

[R26] Graupner V, Alexander E, Overkamp T, Rothfuss O, De Laurenzi V, Gillissen BF, Daniel PT, Schulze-Osthoff K, Essmann F (2011). Differential regulation of the proapoptotic multidomain protein Bak by p53 and p73 at the promoter level. Cell Death Differ.

[R27] Grespi F, Soratroi C, Krumschnabel G, Sohm B, Ploner C, Geley S, Hengst L, Hacker G, Villunger A (2010). BH3-only protein Bmf mediates apoptosis upon inhibition of CAP-dependent protein synthesis. Cell Death Differ.

[R28] Martinou JC, Youle RJ (2011). Mitochondria in apoptosis: Bcl-2 family members and mitochondrial dynamics. Dev Cell.

[R29] Cleland MM, Norris KL, Karbowski M, Wang C, Suen DF, Jiao S, George NM, Luo X, Li Z, Youle RJ (2011). Bcl-2 family interaction with the mitochondrial morphogenesis machinery. Cell Death Differ.

[R30] Dunkle A, Dzhagalov I, He YW (2010). Mcl-1 promotes survival of thymocytes by inhibition of Bak in a pathway separate from Bcl-2. Cell Death Differ.

[R31] Kutuk O, Letai A (2010). Displacement of Bim by Bmf and Puma rather than increase in Bim level mediates paclitaxel-induced apoptosis in breast cancer cells. Cell Death Differ.

[R32] Zhang L, Lopez H, George NM, Liu X, Pang X, Luo X (2011). Selective involvement of BH3-only proteins and differential targets of Noxa in diverse apoptotic pathways. Cell Death Differ.

[R33] Altieri DC (2010). Survivin and IAP proteins in cell-death mechanisms. Biochem J.

[R34] Olsson M, Zhivotovsky B (2011). Caspases and cancer. Cell Death Differ.

[R35] Kumar S (2007). Caspase function in programmed cell death. Cell Death Differ.

[R36] Nicholson DW (1999). Caspase structure, proteolytic substrates, and function during apoptotic cell death. Cell Death Differ.

[R37] Maas C, Verbrugge I, de Vries E, Savich G, van de Kooij LW, Tait SW, Borst J (2010). Smac/DIABLO release from mitochondria and XIAP inhibition are essential to limit clonogenicity of Type I tumor cells after TRAIL receptor stimulation. Cell Death Differ.

[R38] Probst BL, Liu L, Ramesh V, Li L, Sun H, Minna JD, Wang L (2010). Smac mimetics increase cancer cell response to chemotherapeutics in a TNF-alpha-dependent manner. Cell Death Differ.

[R39] Karbowski M, Lee YJ, Gaume B, Jeong SY, Frank S, Nechushtan A, Santel A, Fuller M, Smith CL, Youle RJ (2002). Spatial and temporal association of Bax with mitochondrial fission sites, Drp1, and Mfn2 during apoptosis. J Cell Biol.

[R40] Frank S, Gaume B, Bergmann-Leitner ES, Leitner WW, Robert EG, Catez F, Smith CL, Youle RJ (2001). The role of dynamin-related protein 1, a mediator of mitochondrial fission, in apoptosis. Dev Cell.

[R41] Thomenius M, Freel CD, Horn S, Krieser R, Abdelwahid E, Cannon R, Balasundaram S, White K, Kornbluth S (2011). Mitochondrial fusion is regulated by Reaper to modulate Drosophila programmed cell death. Cell Death Differ.

[R42] Liu X, Hajnoczky G (2011). Altered fusion dynamics underlie unique morphological changes in mitochondria during hypoxia-reoxygenation stress. Cell Death Differ.

[R43] Cereghetti GM, Costa V, Scorrano L (2010). Inhibition of Drp1-dependent mitochondrial fragmentation and apoptosis by a polypeptide antagonist of calcineurin. Cell Death Differ.

[R44] Darding M, Feltham R, Tenev T, Bianchi K, Benetatos C, Silke J, Meier P (2011). Molecular determinants of Smac mimetic induced degradation of cIAP1 and cIAP2. Cell Death Differ.

[R45] Goldsmith KC, Lestini BJ, Gross M, Ip L, Bhumbla A, Zhang X, Zhao H, Liu X, Hogarty MD (2010). BH3 response profiles from neuroblastoma mitochondria predict activity of small molecule Bcl-2 family antagonists. Cell Death Differ.

[R46] Iaccarino I, Martins LM (2011). Therapeutic targets in cancer cell metabolism and death. Cell Death Differ.

[R47] Morizot A, Merino D, Lalaoui N, Jacquemin G, Granci V, Iessi E, Lanneau D, Bouyer F, Solary E, Chauffert B, Saas P, Garrido C, Micheau O (2011). Chemotherapy overcomes TRAIL-R4-mediated TRAIL resistance at the DISC level. Cell Death Differ.

[R48] Kelly PN, Strasser A (2011). The role of Bcl-2 and its pro-survival relatives in tumourigenesis and cancer therapy. Cell Death Differ.

[R49] Affaticati P, Mignen O, Jambou F, Potier MC, Klingel-Schmitt I, Degrouard J, Peineau S, Gouadon E, Collingridge GL, Liblau R, Capiod T, Cohen-Kaminsky S (2011). Sustained calcium signalling and caspase-3 activation involve NMDA receptors in thymocytes in contact with dendritic cells. Cell Death Differ.

[R50] Hughes R, Kristiansen M, Lassot I, Desagher S, Mantovani R, Ham J (2011). NF-Y is essential for expression of the proapoptotic bim gene in sympathetic neurons. Cell Death Differ.

[R51] Tobaben S, Grohm J, Seiler A, Conrad M, Plesnila N, Culmsee C (2011). Bid-mediated mitochondrial damage is a key mechanism in glutamate-induced oxidative stress and AIF-dependent cell death in immortalized HT-22 hippocampal neurons. Cell Death Differ.

[R52] Rufini A, Melino G (2011). Cell death pathology: the war against cancer. Biochemical and biophysical research communications.

[R53] Hao Z, Duncan GS, Chang CC, Elia A, Fang M, Wakeham A, Okada H, Calzascia T, Jang Y, You-Ten A, Yeh WC, Ohashi P, Wang X (2005). Specific ablation of the apoptotic functions of cytochrome C reveals a differential requirement for cytochrome C and Apaf-1 in apoptosis. Cell.

[R54] Leadsham JE, Kotiadis VN, Tarrant DJ, Gourlay CW (2010). Apoptosis and the yeast actin cytoskeleton. Cell Death Differ.

[R55] Chen ZX, Pervaiz S (2010). Involvement of cytochrome c oxidase subunits Va and Vb in the regulation of cancer cell metabolism by Bcl-2. Cell Death Differ.

[R56] Chen ZX, Pervaiz S (2009). BCL-2: pro-or anti-oxidant?. Front Biosci (Elite Ed).

[R57] Joza N, Oudit GY, Brown D, Benit P, Kassiri Z, Vahsen N, Benoit L, Patel MM, Nowikovsky K, Vassault A, Backx PH, Wada T, Kroemer G (2005). Muscle-specific loss of apoptosis-inducing factor leads to mitochondrial dysfunction, skeletal muscle atrophy, and dilated cardiomyopathy. Mol Cell Biol.

[R58] Krammer PH, Behrmann I, Daniel P, Dhein J, Debatin KM (1994). Regulation of apoptosis in the immune system. Curr Opin Immunol.

[R59] Trifunovic A, Larsson NG (2008). Mitochondrial dysfunction as a cause of ageing. J Intern Med.

[R60] Balaban RS, Nemoto S, Finkel T (2005). Mitochondria, oxidants, and aging. Cell.

[R61] Hur JH, Cho J, Walker DW (2010). Aging: Dial M for Mitochondria. Aging (Albany NY).

[R62] Chen YF, Kao CH, Chen YT, Wang CH, Wu CY, Tsai CY, Liu FC, Yang CW, Wei YH, Hsu MT, Tsai SF, Tsai TF (2009). Cisd2 deficiency drives premature aging and causes mitochondria-mediated defects in mice. Genes Dev.

[R63] Liu J, Cao L, Chen J, Song S, Lee IH, Quijano C, Liu H, Keyvanfar K, Chen H, Cao LY, Ahn BH, Kumar NG, Rovira II (2009). Bmi1 regulates mitochondrial function and the DNA damage response pathway. Nature.

[R64] Reilly SM, Bhargava P, Liu S, Gangl MR, Gorgun C, Nofsinger RR, Evans RM, Qi L, Hu FB, Lee CH (2010). Nuclear receptor corepressor SMRT regulates mitochondrial oxidative metabolism and mediates aging-related metabolic deterioration. Cell Metab.

[R65] Rufini A, Niklison-Chirou MV, Inoue S, Tomasini R, Harris IS, Marino A, Federici M, Dinsdale D, Knight RA, Melino G, Mak TW (2012). TAp73 depletion accelerates aging through metabolic dysregulation. Genes & development.

[R66] Sahin E, Colla S, Liesa M, Moslehi J, Muller FL, Guo M, Cooper M, Kotton D, Fabian AJ, Walkey C, Maser RS, Tonon G, Foerster F (2011). Telomere dysfunction induces metabolic and mitochondrial compromise. Nature.

[R67] Trifunovic A, Wredenberg A, Falkenberg M, Spelbrink JN, Rovio AT, Bruder CE, Bohlooly YM, Gidlof S, Oldfors A, Wibom R, Tornell J, Jacobs HT, Larsson NG (2004). Premature ageing in mice expressing defective mitochondrial DNA polymerase. Nature.

[R68] Velarde MC, Flynn JM, Day NU, Melov S, Campisi J (2012). Mitochondrial oxidative stress caused by Sod2 deficiency promotes cellular senescence and aging phenotypes in the skin. Aging (Albany NY).

[R69] Barrientos A (2012). Complementary roles of mitochondrial respiration and ROS signaling on cellular aging and longevity. Aging (Albany NY).

[R70] Trifunovic A, Hansson A, Wredenberg A, Rovio AT, Dufour E, Khvorostov I, Spelbrink JN, Wibom R, Jacobs HT, Larsson NG (2005). Somatic mtDNA mutations cause aging phenotypes without affecting reactive oxygen species production. Proc Natl Acad Sci U S A.

[R71] Fontana L, Partridge L, Longo VD (2010). Extending healthy life span—from yeast to humans. Science.

[R72] Wang C, Maddick M, Miwa S, Jurk D, Czapiewski R, Saretzki G, Langie SA, Godschalk RW, Cameron K, von Zglinicki T (2010). Adult-onset, short-term dietary restriction reduces cell senescence in mice. Aging (Albany NY).

[R73] Choksi KB, Nuss JE, DeFord JH, Papaconstantinou J (2011). Mitochondrial electron transport chain functions in long-lived Ames dwarf mice. Aging (Albany NY).

[R74] Speakman JR, Mitchell SE (2011). Caloric restriction. Mol Aspects Med.

[R75] Nisoli E, Tonello C, Cardile A, Cozzi V, Bracale R, Tedesco L, Falcone S, Valerio A, Cantoni O, Clementi E, Moncada S, Carruba MO (2005). Calorie restriction promotes mitochondrial biogenesis by inducing the expression of eNOS. Science.

[R76] Raffaello A, Rizzuto R (2011). Mitochondrial longevity pathways. Biochim Biophys Acta.

[R77] Zid BM, Rogers AN, Katewa SD, Vargas MA, Kolipinski MC, Lu TA, Benzer S, Kapahi P (2009). 4E-BP extends lifespan upon dietary restriction by enhancing mitochondrial activity in Drosophila. Cell.

[R78] Choudhury M, Jonscher KR, Friedman JE (2011). Reduced mitochondrial function in obesity-associated fatty liver: SIRT3 takes on the fat. Aging (Albany NY).

[R79] Boudina S, Sena S, O'Neill BT, Tathireddy P, Young ME, Abel ED (2005). Reduced mitochondrial oxidative capacity and increased mitochondrial uncoupling impair myocardial energetics in obesity. Circulation.

[R80] Polak P, Cybulski N, Feige JN, Auwerx J, Ruegg MA, Hall MN (2008). Adipose-specific knockout of raptor results in lean mice with enhanced mitochondrial respiration. Cell Metab.

[R81] Ruckenstuhl C, Carmona-Gutierrez D, Madeo F (2010). The sweet taste of death: glucose triggers apoptosis during yeast chronological aging. Aging (Albany NY).

[R82] Weinberger M, Mesquita A, Caroll T, Marks L, Yang H, Zhang Z, Ludovico P, Burhans WC (2010). Growth signaling promotes chronological aging in budding yeast by inducing superoxide anions that inhibit quiescence. Aging (Albany NY).

[R83] Alkhouri N, Gornicka A, Berk MP, Thapaliya S, Dixon LJ, Kashyap S, Schauer PR, Feldstein AE (2010). Adipocyte apoptosis, a link between obesity, insulin resistance, and hepatic steatosis. J Biol Chem.

[R84] Khanna A, Muthusamy S, Liang R, Sarojini H, Wang E (2011). Gain of survival signaling by down-regulation of three key miRNAs in brain of calorie-restricted mice. Aging (Albany NY).

[R85] Bach D, Naon D, Pich S, Soriano FX, Vega N, Rieusset J, Laville M, Guillet C, Boirie Y, Wallberg-Henriksson H, Manco M, Calvani M, Castagneto M (2005). Expression of Mfn2, the Charcot-Marie-Tooth neuropathy type 2A gene, in human skeletal muscle: effects of type 2 diabetes, obesity, weight loss, and the regulatory role of tumor necrosis factor alpha and interleukin-6. Diabetes.

[R86] Jheng HF, Tsai PJ, Guo SM, Kuo LH, Chang CS, Su IJ, Chang CR, Tsai YS (2012). Mitochondrial fission contributes to mitochondrial dysfunction and insulin resistance in skeletal muscle. Mol Cell Biol.

[R87] Kelley DE, He J, Menshikova EV, Ritov VB (2002). Dysfunction of mitochondria in human skeletal muscle in type 2 diabetes. Diabetes.

[R88] Zhao J, Pei G (2011). Why cell reprogramming is functionally linked to aging?. Aging (Albany NY).

[R89] Jasper H, Jones DL (2010). Metabolic regulation of stem cell behavior and implications for aging. Cell Metab.

[R90] Yilmaz OH, Katajisto P, Lamming DW, Gultekin Y, Bauer-Rowe KE, Sengupta S, Birsoy K, Dursun A, Yilmaz VO, Selig M, Nielsen GP, Mino-Kenudson M, Zukerberg LR (2012). mTORC1 in the Paneth cell niche couples intestinal stem-cell function to calorie intake. Nature.

[R91] Dupont-Versteegden EE (2005). Apoptosis in muscle atrophy: relevance to sarcopenia. Exp Gerontol.

[R92] Marzetti E, Lawler JM, Hiona A, Manini T, Seo AY, Leeuwenburgh C (2008). Modulation of age-induced apoptotic signaling and cellular remodeling by exercise and calorie restriction in skeletal muscle. Free Radic Biol Med.

[R93] Gunasekaran U, Gannon M (2011). Type 2 diabetes and the aging pancreatic beta cell. Aging (Albany NY).

